# Causal associations of fatigue and functional outcome after ischemic stroke: a mediation Mendelian randomization study

**DOI:** 10.3389/fneur.2024.1415553

**Published:** 2024-07-25

**Authors:** Ping Jiang, Ying Gao, Leyi Zhang, Li Jiang, Chuanpeng Li

**Affiliations:** ^1^Dongzhimen Hospital, Beijing University of Chinese Medicine, Beijing, China; ^2^Institute for Brain Disorders, Dongzhimen Hospital, Beijing University of Chinese Medicine, Beijing, China

**Keywords:** fatigue, functional recovery, ischemic stroke, mediation Mendelian randomization, lipid metabolites

## Abstract

**Background and objectives:**

Fatigue has been associated with adverse effects on recovery from ischemic stroke based on previous observational research. The purpose of our study was to explore the potential causal association of fatigue with poor functional outcome after ischemic stroke by employing Mendelian randomization (MR).

**Methods:**

A set of instrumental variables, comprising 36 single-nucleotide polymorphisms (SNPs) that are only related to fatigue, were derived from a genome-wide association study (GWAS) that included 449,019 general individuals. The functional outcomes after ischemic stroke were derived from a GWAS (Genetics of Ischemic Stroke Functional Outcome Network) involving 6,021 survivors. Two-sample MR methods were used to assess the causal effect, including inverse variance weighted, MR-Egger, weighted median, simple mode, and weighted mode. In bidirectional MR analysis, the reverse causal association was analyzed using the Wald ratio method. The mediation effects of lipid metabolites were analyzed using two-step MR analysis.

**Results:**

Genetic liability to fatigue was causally associated with the poor functional outcome (modified Rankin Scale ≥3 at 3 months) after ischemic stroke (OR = 4.20, 95%CI [1.11–15.99], *p* < 0.05). However, genetic predicted poor functional outcome after ischemic stroke was not associated with fatigue (OR = 1.00, 95%CI [0.99–1.02], *p* > 0.05). The results of the two-step MR showed that cholesteryl esters to total lipids ratio in large very low-density lipoprotein (VLDL) (ME = −0.13, *p* < 0.05); concentration of very large VLDL particles (ME = −0.13, *p* < 0.05); free cholesterol in large VLDL (ME = −0.13, *p* < 0.05); free cholesterol to total lipids ratio in very large VLDL (ME = −0.22, *p* < 0.05); phospholipids in large VLDL (ME = −0.15, *p* < 0.05); phospholipids in very large VLDL (ME = −0.13, *p* < 0.05); phospholipids to total lipids ratio in large high-density lipoprotein (HDL) (ME = −0.17, *p* < 0.05); total lipids in very large VLDL (ME = −0.14, *p* < 0.05); triglycerides in small VLDL (ME = −0.11, *p* < 0.05); and triglycerides to total lipids ratio in large HDL (ME = −0.10, *p* < 0.05) assumed a pivotal role in mediating the association between fatigue and poor functional outcome after ischemic stroke.

**Conclusion:**

Our study provides evidence supporting the causal association between fatigue and the poor functional outcome after ischemic stroke, which emphasizes the importance of implementing interventions aimed at addressing fatigue. This could offer a therapeutic target to improve recovery after ischemic stroke and warrant exploration in a clinical context. One potential mechanism by which fatigue affects functional outcomes after ischemic stroke is through the action of lipid metabolites.

## 1 Introduction

In 2019, stroke was the third-leading global combined cause of death and disability (1). Over the past two decades, there has been a notable increase in the burden attributed to ischemic stroke (2). The poor functional outcome in patients with ischemic stroke may have multiple underlying causes. However, evidence suggests that it may be associated with the emergence of early neuropsychiatric symptoms, such as insomnia, depression, anxiety or fatigue (3–6). The 2019 update of the Canadian Stroke Best Practice Recommendations (CSBPR) for Mood, Cognition, and Fatigue following Stroke emphasizes early recognition and treatment to avoid these conditions leading to worse long-term outcomes (7). Nevertheless, the causal relationships between fatigue and poor functional outcomes post-ischemic stroke remain contentious and warrant further clarification.

The prevalence of fatigue was reported to be around 48% in stroke survivors (8). Post-stroke fatigue is characterized by a persistent and overwhelming sense of tiredness or exhaustion that is not necessarily correlated with physical activity and does not improve with rest (9–11). After a decade of follow-up, a study found that fatigue was associated with poor functional outcome after stroke (12). Several subsequent observational studies have reported that fatigue might have negative effects on stroke recovery (13–15). Post-stroke fatigue had a significant impact on the possibility of returning to work (16). The current evidence between fatigue and poor functional outcome after ischemic stroke is primarily from observational studies, which could be biased by potential unmeasured confounders and reverse causations (17). Furthermore, randomized controlled trials are unfeasible, which leads to a lack of causal evidence between fatigue and adverse outcomes after stroke. Given the paucity of attention paid by healthcare professionals to post-stroke fatigue (18, 19), based on current correlation evidence, we conducted a causal study on the relationship between fatigue and poor functional outcome after ischemic stroke. In addition, the mechanisms by which fatigue affects functional outcome after ischemic stroke are not yet well understood (20).

Mendelian randomization (MR) uses single nucleotide polymorphisms (SNPs) as instrumental variables for studying the effect of an exposure on the outcomes (21). The MR approach holds the potential to reduce residual confounding because SNPs are randomly distributed during conception and are thus unlikely to be associated with environmental confounders. Furthermore, MR may mitigate the risk of reverse causality bias as SNPs are determined from conception (21, 22). Therefore, if an SNP that modifies susceptibility to fatigue is also linked to functional outcome after stroke, it would add evidence to support that fatigue is of causal relevance in affecting stroke recovery. To our knowledge, the associations between fatigue and functional outcome after ischemic stroke have not been evaluated previously by using MR.

## 2 Materials and methods

### 2.1 Study reporting guidelines and study design

**Figure 1:**
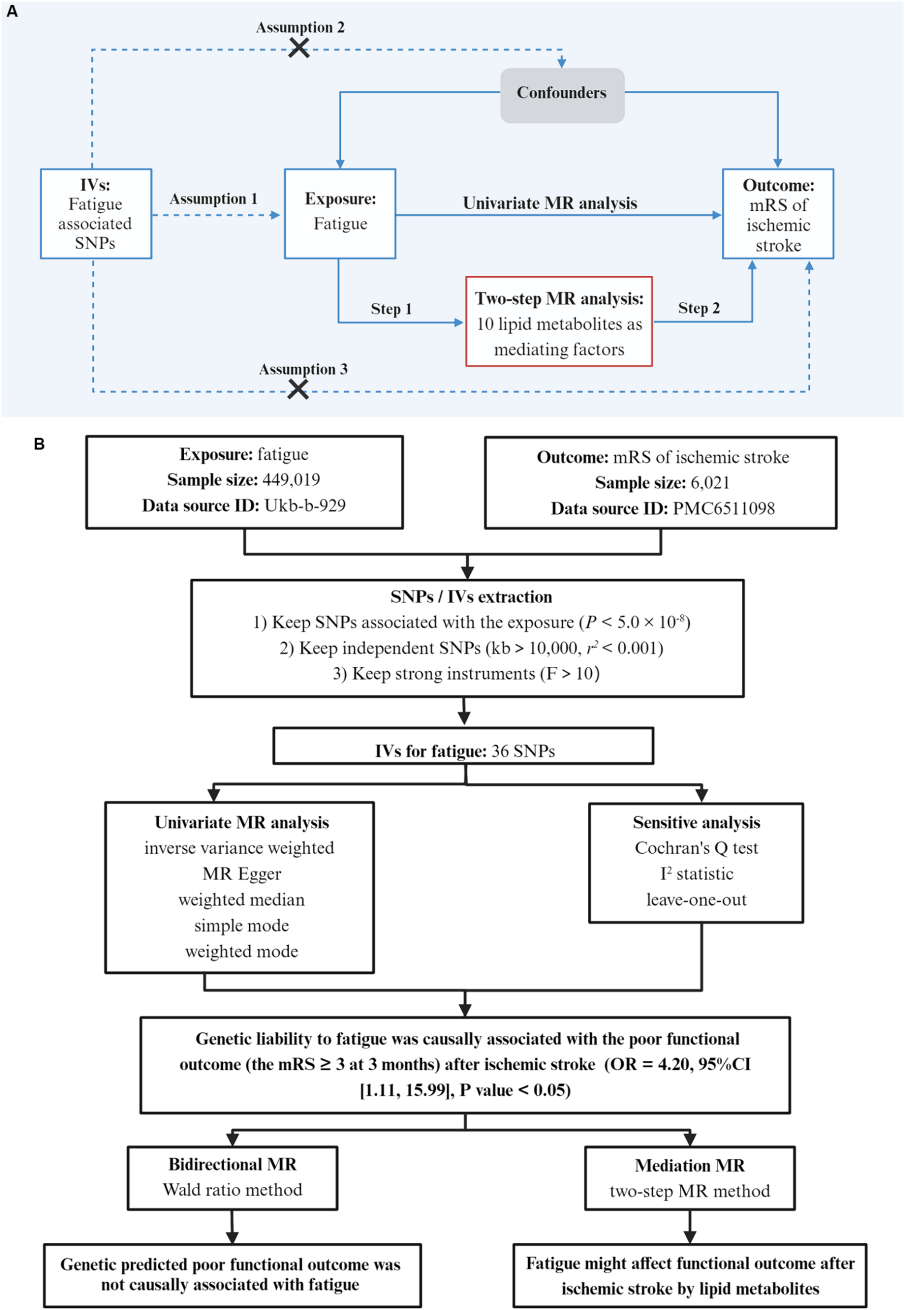
Study design. **(A)** Basic assumptions of Mendelian randomization: Assumption 1 states that SNPs are closely associated with the exposure; Assumption 2 states that SNPs are not associated with any potential confounders; Assumption 3 states that SNPs are only linked to the outcome through exposure. Workflow of the two-step MR analysis. Step 1 estimated the causal effect of fatigue on lipid metabolites, and step 2 assessed the causal effect of lipid metabolites on the poor outcome after ischemic stroke. **(B)** The overall flow chart of this study and the main results. The outcome was the functional outcome after ischemic stroke which was evaluated by the modified Rankin Scale (mRS) at 3 months. The mRS ≥ 3 was considered to be a poor functional outcome. IVs, instrumental variables; SNPs, single nucleotide polymorphism; MR, Mendelian randomization.

We used two-sample MR and publicly available datasets to investigate the causal associations of fatigue and the poor functional outcome after ischemic stroke. Informed consent was obtained and ethical review was sough in all of the original studies (23, 24). All data were available and openly accessible to the public. The schematic diagram of the research design is shown in Figure 1. The research was reported following the STROBE-MR statement (25).

### 2.2 Data sources and ascertainment of included traits in this study

Instrumental variables for fatigue were derived from a genome-wide association study (GWAS) that aimed to investigate the genetic contribution to self-reported tiredness/fatigue (26). Fatigue was evaluated using self-reported frequency of tiredness/lethargy in the last 2 weeks (Do not know; not at all; several days; more than half the days; nearly every day), which was asked as part of the Mental Health Questionnaire that consists of items from the Patient Health Questionnaire (27). The GWAS study was based on a UK Biobank sample that had 449,019 general individuals in total^1^ in Table 1.

**Table 1:** Data sources of included traits in this study.

Traits	Data source	Sample size	Ethnic origin	ID
Fatigue	UKB	449,019	European	Ukb-b-929
Ischemic stroke outcome	GISCOME network	6,021	Mixed	PMC6511098

GISCOME, Genetics of Ischemic Stroke Functional Outcome; UKB, UK Biobank.

The GWAS data on functional outcomes after ischemic stroke comprises the 3-month modified Rankin Scale (mRS) scores in 6,021 patients with ischemic stroke from 12 studies in Europe, the United States and Australia (Supplementary Table S1) (23). The data can be accessed via the following website: https://cd.hugeamp.org/dinspector.html?dataset=GWAS_GISCOME_eu. The mRS score was divided into two groups (0–2 and 3–6) as a dichotomous variable. The analysis was adjusted for age, sex, ancestry, and baseline stroke severity (as measured by the National Institutes of Health Stroke Scale at 0–10 days after stroke onset).

### 2.3 Selection of instrumental variables

A valid genetic-variation instrumental variable (IV) must satisfy three core assumptions: the associativity assumption, i.e., the selected IV must be significantly associated with the exposure factor; independence assumption, i.e., the IV must not be significantly related to potential confounders that may affect the exposure or outcome; exclusivity limitation, i.e., the IV can only affect the outcome through the path of “IV → exposure → outcome” (22).

When screening for fatigue-related IVs, we followed the core assumptions. First, we selected SNPs with statistically significant associations in GWAS, and their *p*-values needed to be less than 5 × 10^−8^. Second, we ensured that the selected SNPs satisfied the condition of $r^{2}<0.001$ , and that any two physical distances between genes were greater than 10,000 kb, taking into account linkage disequilibrium. IVs were extracted from GWAS outcome data based on the selected SNPs in Supplementary Table S2. *F*-statistics were calculated to assess weak IV bias. When *F* < 10, it indicates that the genetic variation used is a weak IV, which may have a certain bias on the results, so it should be removed to avoid affecting the results (28). The formula for calculating the F-statistic is as follows:


$$F=\frac{N-k-1}{k}\times \frac{R^{2}}{1-R^{2}}$$

where *N* is the sample size, *k* is the number of IVs used, and *R*^2^ reflects the extent to which the IVs explain the exposure. *R*^2^ = 2 × (1 − MAF) × MAF × 2*β*, where MAF is the minimum allele frequency and *β* is the allele effect size.

### 2.4 Estimation of MR causal effects

A range of two-sample MR methods were used to assess the causal effect of exposure on outcomes, including inverse-variance weighted (IVW), Mendelian randomization-Egger (MR-Egger), weighted median, simple mode, and weighted mode. When there was only one instrumental variable, the Wald ratio method was used to estimate the causal effect. Because the IVM method does not consider the existence of intercept terms in regression and used the reciprocal of the outcome variance as the weight for fitting, it is generally superior to other MR analysis methods (29). Therefore, in the absence of pleiotropy, regardless of the existence of heterogeneity, this study used the IVW method as the main MR analysis method, and the other four methods were used as supplements (the IVW random effect model was used when heterogeneity exists). When pleiotropic effects existed, the results were calculated using the MR-Egger method.

The mediation effects of lipid metabolites were analyzed using two-step MR analysis (Supplementary Table S3) (30). In the first step, IVs for fatigue were used to deduce the causal impact of this exposure on lipid metabolites. In the second step, IVs for lipid metabolites were used to determine the causal effect of the potential mediator on poor functional outcome after ischemic stroke. In instances where evidence suggested that fatigue influenced the mediator, which in turn influenced the functional outcome after ischemic stroke, we utilized the “product of coefficients” method to assess the indirect effect of fatigue on poor functional outcome after ischemic stroke via lipid metabolites. Standard errors for the indirect effects were derived by using the delta method.

### 2.5 Sensitivity analysis and statistical analysis

All data calculations and statistical analysis were performed using R programming,^2^ mainly using the two-sample MR package to carry out the analysis (31). Cochran’s *Q*-test and leave-one-out analysis were used to test the reliability and robustness of the evaluation results. MR Egger intercept method was used for genetic pleiotropy testing. The evaluation indicators were odds ratio (OR) and 95% confidence interval (CI). All statistical *p* values were two-sided tests. *p* < 0.05 was considered to indicate statistically significant differences. To ensure that the methods employed in this study are fully documented and accessible for replication, all relevant code files were uploaded to a public repository.^3^

## 3 Results

### 3.1 Screening of IVs

The number of IVs of fatigue was 36 (Supplementary Table S2). The *F*-test statistics all of these instrumental variables were ≥10, indicating that most of the SNPs screened in this study were free from weak instrumental bias.

### 3.2 MR causal effect of fatigue and functional outcome of ischemic stroke

**Figure 2:**
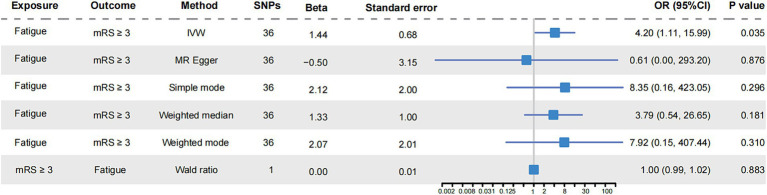
Mendelian randomization analysis of fatigue and functional outcome after ischemic stroke. The forest plot showed the causal analysis results of various MR models on fatigue and functional outcome of ischemic stroke. The effect evaluation value was displayed by OR and 95% CI. It also showed the number of instrumental variables used in each model, as well as the calculated Beta value and standard error. mRS, the modified Rankin Scale; SNPs, single nucleotide polymorphisms; OR, odds ratio; CI, confidence interval; IVW, inverse-variance weighted.

**Figure 3:**
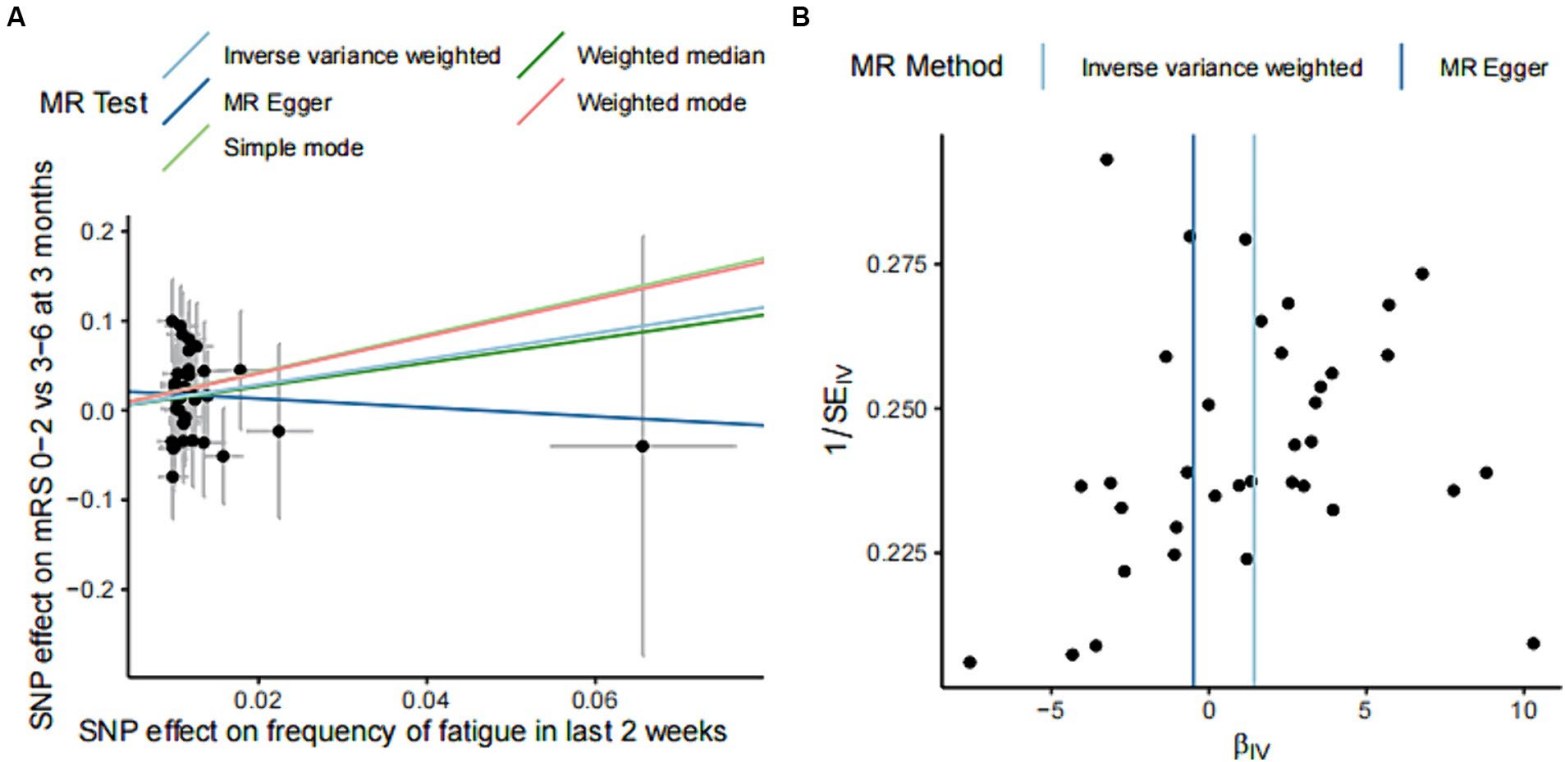
Effect estimation of five models of MR analysis of fatigue and functional outcome after ischemic stroke and heterogeneity test funnel plot. **(A)** The scatter plot shows the estimation of the causal effect of fatigue and poor functional outcome of ischemic stroke. The slope of the line indicates the causality predicted by different models. **(B)** Funnel plots showing the estimation of the causal effect of fatigue on each instrumental variable for poor outcome, with the causal effect estimates from the Inverse variance weighted and MR Egger models labeled with a straight line on the plots. SNPs, single nucleotide polymorphisms; MR, Mendelian randomization.

**Figure 4:**
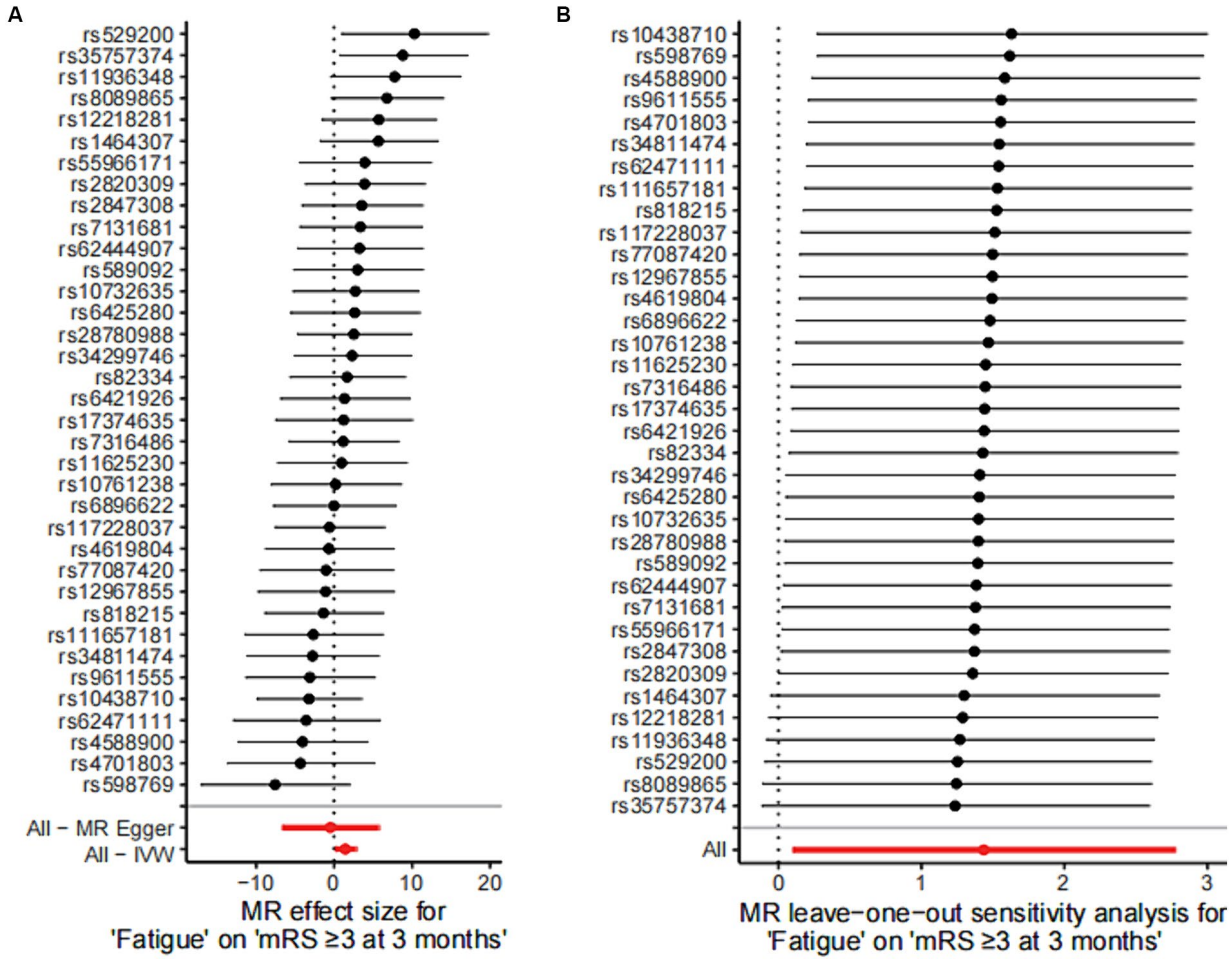
Mendelian randomized instrumental variable effects of fatigue and functional outcome after ischemic stroke and leave-one-out analysis. **(A)** Mendelian randomization analysis of the effect value of each instrumental variable on functional outcome after stroke was shown in a forest plot. **(B)** Estimates of the causal effect after excluding SNPs one by one (leave-one-out analysis) were shown in a forest plot. Estimates of the causal effect of the MR model were marked in red in the figure. MR, Mendelian randomization; IVW, inverse variance weighted.

In IVM model, there was a significant causal relationship between fatigue and the poor functional outcome (mRS ≥ 3) after ischemic stroke (OR = 4.20, 95%CI [1.11–15.99], *p* < 0.05) (Figure 2). The MR analysis across the five models revealed that while most models provided consistent direct estimates, one model produced inconsistent estimates, and there were notable discrepancies in the slopes observed (Figure 3A). The effect size of each IV from MR analysis on the poor stroke outcome is shown as a forest plot (Figure 4A). We further explored the reverse causal association as shown in Figure 2, There was only one IV for mRS ≥ 3 after ischemic stroke, so it was analyzed using the Wald ratio method, which showed that the poor functional outcome was not associated with fatigue (OR = 1.00, 95%CI [0.99–1.02], *p* > 0.05).

### 3.3 Sensitivity analysis

Significant results were tested for heterogeneity using the Cochran *Q* test and the *I*^2^ statistic. The results showed that there was no heterogeneity in the results of the MR analysis (Cochran *Q p*-value = 0.715). Funnel plots of the IVs for fatigue and functional outcome after ischemic stroke showed an essentially symmetrical distribution of the scatter of causal association effects, suggesting that there was no potential bias in the results (Figure 3B).

The statistical hypothesis test *p*-value of the intercept term was 0.534, and the intercept was close to 0 (Egger intercept = 0.023), indicating that the causal inference in this study was not affected by horizontal pleiotropy.

Sensitivity analysis were conducted by using the leave-one-out approach, and no change was detected in the significance of the estimate of the negative effect of fatigue on stroke, suggesting that the results were stable (Figure 4B).

### 3.4 Mediation effects of lipid metabolites

High-density lipoprotein (HDL) and very-low-density lipoprotein (VLDL) are pertinent to cardiovascular health. The results of the two-step MR showed that cholesteryl esters to total lipids ratio in large VLDL (ME = −0.13, *p* < 0.05); concentration of very large VLDL particles (ME = −0.13, *p* < 0.05); free cholesterol in large VLDL (ME = −0.13, *p* < 0.05); free cholesterol to total lipids ratio in very large VLDL (ME = −0.22, *p* < 0.05); phospholipids in large VLDL (ME = −0.15, *p* < 0.05); phospholipids in very large VLDL (ME = −0.13, *p* < 0.05); phospholipids to total lipids ratio in large HDL (ME = −0.17, *p* < 0.05); total lipids in very large VLDL (ME = −0.14, *p* < 0.05); triglycerides in small VLDL (ME = −0.11, *p* < 0.05); and triglycerides to total lipids ratio in large HDL (ME = −0.10, *p* < 0.05) assumed a pivotal role in mediating the association between fatigue and poor functional outcome after ischemic stroke (Table 2).

**Table 2:** Mediation effects of lipid metabolites on the causal association between fatigue and the poor functional outcome after ischemic stroke.

Mediator	ME (95%CI)	PTE	Effect 1	Effect 2
Cholesteryl esters to total lipids ratio in large VLDL	−0.13 (−0.25, −0.02)	−9.03	0.40	0.26
Concentration of very large VLDL particles	−0.13 (−0.25, −0.01)	−9.03	0.43	0.25
Free cholesterol in large VLDL	−0.13 (−0.24, −0.03)	−9.03	0.39	0.32
Free cholesterol to total lipids ratio in very large VLDL	−0.22 (−0.37, −0.06)	−15.28	0.49	0.31
Phospholipids in large VLDL	−0.15 (−0.26, −0.04)	−10.42	0.41	0.34
Phospholipids in very large VLDL	−0.13 (−0.24, −0.02)	−9.03	0.40	0.28
Phospholipids to total lipids ratio in large HDL	−0.17 (−0.31, −0.03)	−11.81	0.47	0.26
Total lipids in very large VLDL	−0.14 (−0.26, −0.03)	−9.72	0.43	0.30
Triglycerides in small VLDL	−0.11 (−0.20, −0.01)	−7.64	0.38	0.27
Triglycerides to total lipids ratio in large HDL	−0.10 (−0.19, −0.02)	−6.94	0.36	0.22

ME, mediation effect; CI, confidence interval; PTE, percentage of total effect; Effect 1, fatigue to lipid metabolites; Effect 2, lipid metabolites to the poor functional outcome after ischemic stroke; HDL, high-density lipoprotein; VLDL, very-low-density lipoprotein.

## 4 Discussion

The results of this MR analysis showed that genetically predicted fatigue was causally associated with poor functional outcome after ischemic stroke. Lipid metabolites might play a significant mediator in this association.

To our knowledge, this is the first study to examine the causal relation of fatigue to poor functional outcome after ischemic stroke. Previous observational epidemiologic studies investigating the relationship between fatigue and outcomes after ischemic stroke found consistent negative effects, despite using different evaluation tools for stroke outcomes and fatigue. Fatigue during the acute phase following the first-ever stroke was associated with activity limitations at the 18-month follow-up (32, 33). In a study of 377 patients, Cox regression analysis showed that post-stroke fatigue was associated with higher mortality (34). In a study of 218 patients with ischemic stroke, fatigue severity was associated with poorer activities of daily living and health-related quality of life (6). Fatigue was also associated with functional difficulties following arterial ischemic stroke in a pediatric population (35). The scores of the Functional Independence Measure at discharge were significantly lower in the fatigue group than in the non-fatigue group (36). Stroke survivors with fatigue had improvements in their quality of life and radiological characteristics after reducing fatigue levels (37–39). In general, combining previous research results on correlation, our study reinforces the causal association between fatigue and poor functional outcome after ischemic stroke.

Bidirectional MR analysis was performed to boost the strength of the evidence for the causal pathway from fatigue to adverse outcomes following ischemic stroke. The lack of reverse causality observed—where genetically predicted poor functional outcome was not associated with fatigue—suggested that the direction of causality likely originates from fatigue impacting stroke recovery rather than the contrary. Potential explanations for this finding include the temporal precedence of fatigue as a symptom, which often manifests early in the course of recovery and can persist long-term, affecting rehabilitation efforts and overall functional status (32). Unlike transient physiological responses that are directly linked to the acute event of stroke, fatigue may represent a more chronic condition influenced by psychological, neurological, and metabolic factors that are less likely to be directly affected by immediate post-stroke outcomes. This highlights the importance of addressing fatigue as an independent factor in the management of ischemic stroke recovery.

Fatigue might contribute to the poor functional outcome after ischemic stroke because of dysregulation of lipid metabolism. Brain tissue damage is a well-established occurrence after ischemic stroke, and 48% stroke survivors experience symptoms of fatigue (8). Research suggests that individuals who experienced fatigue after ischemic stroke might have reduced excitability in the motor cortex and an increased perception of limb heaviness (40, 41). This might be due to decreased excitation of corticospinal output and its facilitatory synaptic inputs from cortical and subcortical regions (42). Stroke survivors who experience fatigue tend to engage in less physical activity and spend more time in a bedridden state, leading to unhealthy lifestyles (43, 44). Lipid metabolism disorders could result from lifestyle changes. Therefore, considering the established link between lipid metabolic abnormalities and negative outcomes in ischemic stroke, it is plausible that fatigue affects post-stroke recovery by affecting lipid metabolism.

The strength of the study is the MR analysis design is based on large-scale samples. MR analysis is a technique that uses genetic variants to estimate the causal effect of exposure on disease. Potential bias is greatly reduced because genetic variation is not associated with other confounding factors like lifestyle, diet, and behavior, which may influence the results from observational studies (45, 46). MR analysis robustly minimizes biases from early neuropsychiatric symptoms including insomnia, depression, and anxiety, thereby offering a clearer perspective on causal relationships. MR analysis can also avoid reverse causation, as genetic variation is assigned at conception. The nature of the MR analysis design is less prone to potential unmeasured confounding and reverse causation and can strengthen the evidence for causal inference (21). In addition, by using the two-sample MR approach, we could test the effort of fatigue based on large-scale samples. This study used five MR analysis methods and ensured consistent results. Moreover, the reverse causal relationship was verified through bidirectional MR analysis. This methodology bolsters our confidence in the causal inference that fatigue influences post-stroke recovery. Given these findings, we advocate for the inclusion of fatigue as a crucial variable in predictive models for acute ischemic stroke prognosis (47). Incorporating fatigue into these models could potentially enhance their predictive accuracy and clinical utility, complementing the contribution of our research to understanding the causal link between fatigue and post-stroke functional outcomes, thereby opening further avenues to explore medicinal genes that may facilitate improved stroke recovery strategies.

Although our study provides valuable guidance for clinical practice and significantly advances the understanding of biological mechanisms linking fatigue with poor outcome after ischemic stroke, we acknowledge that the study also has some limitations. First, the investigation of fatigue was limited to individuals of European ancestry, which may limit the generalizability of our findings to other ethnic populations. Although multiple strategies were employed to address pleiotropy concerns, it is challenging to completely exclude potential violations of IV assumptions. Additionally, this work might not have captured all lipid metabolites that mediate the relationship between fatigue and stroke outcomes, nor exhaustively explored alternative intermediary pathways such as inflammation, mitochondrial autophagy, and amino acid metabolism. Future research requires more comprehensive lipid metabolomics or other high-throughput sequencing analyses based on large-scale cohorts to improve the accuracy of these investigations.

## 5 Conclusion

To our knowledge, this study is the first to establish a causal relationship between fatigue and post-ischemic stroke dysfunction by using MR. Our findings indicate that fatigue significantly influences stroke recovery. Given the potential link between lipid metabolites and both stroke recovery and fatigue, these metabolites could be the focus of future intervention approaches. Despite limitations such as potential violations of IV assumptions and ethnic constraints, the research design effectively mitigates confounding factors and reverse causality. Future research should examine the generalizability of these findings across different populations and explore the specific mechanisms by which lipid metabolites influence this relationship. In conclusion, assessing and managing fatigue is crucial for improving functional outcomes in patients after ischemic stroke.

## Data availability statement

The original contributions presented in the study are included in the article/Supplementary material, further inquiries can be directed to the corresponding author.

## Ethics statement

Ethical review and approval was not required for the study on human participants in accordance with the local legislation and institutional requirements. Written informed consent from the patients/participants or patients/participants’ legal guardian/next of kin was not required to participate in this study in accordance with the national legislation and the institutional requirements.

## Author contributions

PJ: Data curation, Methodology, Writing – original draft, Writing – review & editing. YG: Supervision, Writing – review & editing. LZ: Data curation, Writing – original draft. LJ: Data curation, Writing – original draft. CL: Data curation, Writing – original draft.

## Footnotes

^1^ID: ukb-b-929, https://gwas.mrcieu.ac.uk/datasets/ukb-b-929/.

^2^https://www.r-project.org/, version 4.3.0.

^3^ https://github.com/TinaJiangp/MR-fatigue-to-strokeoutcome
